# A hybrid effectiveness-implementation cluster randomized trial of group CBT for anxiety in urban schools: rationale, design, and methods

**DOI:** 10.1186/s13012-016-0453-z

**Published:** 2016-07-12

**Authors:** Ricardo Eiraldi, Muniya S. Khanna, Abbas F. Jawad, Jessica Fishman, Henry A. Glick, Billie S. Schwartz, Jaclyn Cacia, Abraham Wandersman, Rinad Beidas

**Affiliations:** 1 The Children’s Hospital of Philadelphia, 3535 Market Street, Rm. 1474, Philadelphia, PA 19104 USA; 2 Department of Pediatrics, University of Pennsylvania Perelman School of Medicine, 3400 Civic Center Boulevard, Philadelphia, PA 19104 USA; 3 Department of Psychiatry, University of Pennsylvania Perelman School of Medicine, 3535 Market Street, Philadelphia, PA 19104 USA; 4 Department of Biostatistics and Epidemiology, University of Pennsylvania, 3535 Market Street, Philadelphia, PA 19104 USA; 5 Wharton School, University of Pennsylvania, 3620 Locust Walk, Philadelphia, PA 19104 USA; 6 Leonard Davis Institute of Health Economics, University of Pennsylvania, 3641 Locust Walk # 210, Philadelphia, PA 19104 USA; 7 Department of Psychology, University of South Carolina-Columbia, 1512 Pendleton Street, Barnwell College, Suite #220, Columbia, SC 29208 USA

**Keywords:** Urban schools, Capacity building, Effectiveness, Implementation, Hybrid trial, Co-location model, Group cognitive behavioral therapy, Anxiety disorders

## Abstract

**Background:**

Schools present a context with great potential for the implementation of psychosocial evidence-based practices. Cognitive behavioral therapy (CBT) is an evidence-based practice that has been found to be very effective in treating anxiety in various community settings, including schools. Friends for Life (FRIENDS) is an efficacious group CBT protocol for anxiety. Unfortunately, evidence-based practices for anxiety are seldom employed in under-resourced urban schools, because many treatment protocols are not a good fit for the urban school context or the population, existing behavioral health staff do not receive adequate training or support to allow them to implement the treatment with fidelity, or school districts do not have the resources to contract with external consultants. In our prior work, we adapted FRIENDS to create a more culturally sensitive, focused, and feasible CBT protocol for anxiety disorders (CBT for Anxiety Treatment in Schools (CATS)).

**Methods/design:**

The aim of this 5-year study is to evaluate both the effectiveness of CATS for urban public schools compared to the original FRIENDS as well as compare the implementation strategies (train-the-trainer vs. train-the-trainer + ongoing consultation) by conducting a three-arm, parallel group, type 2 hybrid effectiveness-implementation trial in 18 K-8 urban public schools. We will also assess the cost-effectiveness and the mediators and moderators of fidelity. Ninety therapists, 18 agency supervisors, and 360 children will participate. The interactive systems framework for dissemination and implementation guides the training and support procedures for therapists and supervisors.

**Discussion:**

This study has the potential to demonstrate that agency therapists and supervisors who have had little to no prior exposure to evidence-based practices (EBPs) can implement an anxiety disorder EBP with fidelity. Comparisons of the implementation strategies would provide large urban mental health systems with data to make decisions about the adoption of EBPs.

**Trial registration:**

ClinicalTrials.gov, NCT02651402

## Background

Anxiety disorders (i.e., separation anxiety disorder (SAD), generalized anxiety disorder (GAD), and social anxiety disorder (SoAD)) affect up to 13 % of the child population [1], making them among the most common childhood conditions [1]. Anxiety disorders are highly prevalent among inner city school children and often go unidentified and untreated [2]. Children with these disorders are more likely than their peers to have problems with social, peer and parent-child relations [3], academic achievement [4], school refusal [5], and future socio-emotional adjustment [6]. School factors, such as peer problems, academic pressures, and school violence, can contribute to and exacerbate symptoms [7]. The purpose of this study is to (a) test the effectiveness of two versions of a treatment protocol for anxiety disorders in urban schools, (b) test the effectiveness of two implementation strategies (train-the-trainer and an enhanced version of train-the-trainer), and (c) assess mediators and moderators of the type of support on implementation fidelity.

### Mental health services in urban schools

Public schools have become a common setting for the delivery of mental health services to children and may be the ideal context through which to narrow service disparities [8]. The school is a convenient location where services can often be provided at little or no cost to families [8]. Benefits of providing evidence-based practices (EBPs) in schools include the ability to implement interventions in the very environment in which most symptoms are triggered [9] and to incorporate protocol-specific interventions, with peer and teacher involvement, as needed for generalizability [10].

### EBPs for anxiety disorders

Individual and group cognitive behavioral therapies (CBTs) have been shown to be highly effective for the treatment of anxiety in youth [11, 12]. Group cognitive behavioral therapy (GCBT) requires fewer resources than individual CBT because a single therapist can treat several children at once, thus making it less expensive for use in under-resourced settings such as urban schools. Friends for Life (FRIENDS), a GCBT program, is an effective program for the prevention and treatment of anxiety disorders in children as evidenced by a meta-analysis [13] on school-based interventions for at risk [14] and clinically anxious youth [15, 16]. We have used this program in a prior research study and found it to be effective [17]. However, we also found a number of implementation problems (e.g., poor cultural fit, low feasibility for under-resourced school settings) that could impede widespread adoption in the urban school context.

#### The critical implementation problems

##### Program adaptation

Treatments that are developed and tested for efficacy in highly controlled research settings often do not fit into *real-world* contexts [18]. As a result, most effective treatments in health and mental health settings need to be adapted for new contexts [19, 20]. Lee and colleagues developed a planned adaptation (PA) to address the tension between “implementing programs with fidelity and the need to tailor programs to fit the target population” [21, p. 290]. PA orients the provider to the program and its theoretical orientation. A central tenet of PA is that adaptation must be conducted without altering the program’s core components [21]. PA helps the provider identify components of the program that can be modified and provides a process for conducting adaptations and a direction on how to develop evaluation strategies [21]. We applied PA to FRIENDS in order to make it more feasible for implementation in co-location settings with low-income urban children. We made changes to the language, cultural fit, methods, number of sessions, and activities in the treatment manual, which resulted in additions and substitutions in these areas. This resulted in a briefer, more engaging, and culturally sensitive protocol (CBT for Anxiety Treatment in Schools (CATS)) while still maintaining the five essential components [12, 22] of CBT for anxiety treatment, with emphasis on exposure.

#### Training support

Substantial resources have been dedicated to the dissemination and implementation (D&I) of mental health EBPs [23]. Key to that effort has been the identification of strategies for improving internal capacity (i.e., ability to train and supervise therapists using internal resources) of community-based agencies [24, 25]. Studies have shown that effective training models include initial workshops for therapists that are followed up with supervision, which often includes coaching and performance feedback [26, 27]. An important challenge facing publicly funded mental health agencies is finding cost-effective strategies for supporting supervisors. Most community mental health agencies, where low-income urban children receive services, do not have the resources to pay for ongoing expert direct consultation for therapists who implement EBPs [27]. As such, finding time- and cost-effective alternative strategies for supporting therapists would have a substantial public health impact [27]. An important feasibility question is “Can internal clinical supervisors who are not familiar with EBPs provide effective supervision to therapists after participating in training workshops or do they need more extensive support?” A mostly self-sustaining system as previously described would likely be more practical and less expensive but perhaps lead to fewer positive child outcomes compared to a system where supervisors are provided extended expert consultation.

Two alternative, and potentially cost-effective, strategies are the *cascade* or *train-the-trainer* (TT) approaches, by which agency supervisors are trained to conduct effective supervision, and then go on to train therapists; and a modified train-the-trainer approach by which supervisors receive training *plus* extended consultation (TT+) on conducting effective supervision. TT has been used with adult mental health populations. Two pilot studies using this approach have reported improved therapist knowledge and improved client behavior [28, 29], but it is not known whether these findings could generalize other settings or populations [27]. TT+ has been used by Bruce Chorpita and colleagues with agency supervisors who support therapists in the implementation of modular individual CBT [25]. Highlights of this approach include initial training, biweekly or monthly phone consultations with supervisors, and a supervisor promotion review [25]. This modified train-the-trainer approach has been found to lead to moderate outcome effect sizes [25]. Our study would extend this approach by (a) measuring clinical and implementation outcomes (fidelity and cost), (b) testing the support strategies in a co-location school context, (c) using GCBT instead of individual CBT, and (d) assessing mediators and moderators of fidelity.

### Implementation framework

Our implementation framework is the interactive systems framework (ISF; [30, 31]). ISF was developed to bridge the research-to-practice delay in health and mental health care. It is intended to be a “heuristic for understanding key systems, key functions, and key relationships relevant to the dissemination and implementation process” [30, p. 179]. ISF is composed of three interrelated systems: the synthesis and translation system, the support system, and the delivery system. The function of the synthesis and translation system is to distill information innovations and prepare them for implementation by service providers. The support system supports the work of those who put the innovation into practice. The primary function of the delivery system is the implementation of innovations in real-world settings [30]. We will use the support system and the delivery system because they provide a roadmap for the guiding implementation of EBPs by therapists and supervisors with the support of researchers.

### Mediators and moderators of implementation support

We recognize that the implementation of EBPs, specifically fidelity [32], can be altered by the characteristics of the organization implementing the innovation and the characteristics of the providers (e.g., [33]). We will assess the role of intentions because this construct has been shown to be a proximal determinant of treatment fidelity when practitioners have the ability to act on their intentions [34, 35]. Empirically successful theories of behavior change (including the theory of reasoned action, theory of planned behavior, the integrated model, and others) [36] posit that intention is a proximal determinant of behavior, where intentions influence behavior to the extent that one has the ability (i.e., an enabling environment/service context) to act on them. It is also important to study the role of intentions and the service context environment because these constructs are modifiable. Regardless of the specific results, by testing the hypothesized relationships, these findings will directly inform the design of future, targeted interventions. In particular, the findings will indicate whether interventions should focus on strengthening intentions and/or helping therapists act on them. One or both approaches may be appropriate.

### Service context

Currently, 104,803 children in grades K-8 attend school in the School District of Philadelphia. The Department of Behavioral Health and Intellectual Disability Services (DBHIDS) for the city of Philadelphia oversees the community mental health agencies that provide services to Medicaid recipients. Approximately 20 of these agencies provide school therapeutic services (STS) using a co-location model (i.e., agencies provide services in the schools). Community Behavioral Health, a non-profit corporation that manages Medicaid funds for the city of Philadelphia, is the payer for these services. Community Behavioral Health encourages agencies to use group-based EBPs to serve more children and reduce costs [37]. However, EBPs are rarely used in the school setting, in part, because agencies often do not have the expertise to train and supervise therapists in the delivery of EBPs [38]. Reportedly, 55 % of school districts in the USA use the co-location model, and it is estimated that this number is much higher in large urban settings [39]. Thus, the results of this study and procedures for supporting therapists could generalize to most urban school districts [40].

## Methods/design

Curran and colleagues [41] have proposed three hybrid designs to galvanize the translation of efficacious treatments to enhance their public health impact. These research designs are thought to facilitate “more rapid translational gains, more effective implementation strategies and [yield] more useful information for decision makers” [41, p. 217]. In the type II design, effectiveness and implementation have equal importance. Type II effectiveness-implementation hybrid studies are most appropriate for treatments that have a strong record of efficacy and effectiveness, such as FRIENDS [41, 42].

In the present study, we will use a three-arm, parallel group, type 2 hybrid (effectiveness-implementation) design [41]. The three intervention groups are as follows: (A) FRIENDS with TT implementation strategy (supervisors participate in a training workshop and a booster session on conducting supervision); (B) adapted FRIENDS (CATS) with TT implementation strategy; and (C) CATS using the TT+ implementation strategy (supervisors participate in a training workshop and receive further ongoing consultation on conducting supervision). The effectiveness assessment in the trial will compare FRIENDS vs. CATS (different treatments); the implementation assessment in the trial will compare CATS with TT vs. CATS with TT+ (same treatment, different implementation strategies).

Currently, there are 20 agencies that provide STS in 109 schools in Philadelphia. In order to be eligible for the study, agencies will need to be currently serving schools in economically distressed areas of the city (≥90 % students eligible for free lunch) and have at least two clinical supervisors that supervise STS therapists. Eight agencies meet these criteria and have agreed to participate in the study; these agencies serve 65 schools. Eighteen schools (28 %) from the eligible 65 schools will be randomly selected and invited to participate in the study. The 18 schools and their corresponding agency therapists will be randomly assigned to conditions A, B, or C; therefore, each condition will have six schools. Randomization will be stratified into two categories according to school size: schools with more than 800 students and schools with fewer than or equal to 800 students. The stratification is used to ensure equal distribution of similar-sized schools between the groups. The study biostatistician will produce the randomization lists.

### Participants

Participants will include approximately 90 therapists providing mental health services in schools, a minimum of 18 supervisors, and 360 students. Therapists will participate for 1 year; however, no time limit is placed on supervisors during this study timeframe. The unit of analysis by aim is presented in Table 1. We will have 30 therapists and 120 children per condition. Each therapist will implement one child group for data collection purposes for the course of the trial but will be free to continue to implement child groups outside the research project. This will help us manage resources for data collection as well as minimize the effects of differences in therapist experience and therapist dropout on outcomes.

**Table 1:** Units of analysis

Aim 1—effectiveness (A vs. B)	Aim 2—implementation (B vs. C)	Aim 3—mediators and moderators
• 120 children per treatment protocol, FRIENDS, CATS (240 total)	• 30 therapists per implementation strategy (60 total) • 120 children per implementation strategy (240 total)	• 30 therapists per implementation strategy (90 total)

#### Inclusion criteria

Any agency clinical supervisor with a master’s degree or higher in a mental health field can participate as a supervisor. Any STS therapist with a master’s degree or higher, who provides services in 1 of the 18 participating schools, can serve as a therapist. Any child in grades 4–8, already enrolled in the STS program at their school, and who meets the screening and diagnostic criteria will be considered eligible to participate. The screening criterion is a total score ≥25 on Screen for Child Anxiety Related Disorders (SCARED) [43] completed by a parent or teacher. Children can be enrolled if they are *at risk* for or meet diagnostic criteria for a primary SAD, GAD, or SoAD based on Anxiety Disorders Interview Schedule for Children for DSM-5, parent version (ADIS-5-P; [44]), Spanish version [45]. Children with comorbid conditions of lesser severity than the three target disorders based on ADIS-5-P will also be eligible to participate.

#### Exclusion criteria

Supervisors, therapists, and students not involved or enrolled in STS; children whose primary diagnosis is not SAD, GAD, or SoAD; those with classification of “intellectual disability;” children who have diagnoses that make participation in the study clinically inappropriate (i.e., current substance abuse disorder, psychotic, or autism spectrum disorders, based on school records) because they would be unlikely to benefit from GCBT [46]; or who present at an acute risk to themselves or others, will be excluded.

#### Setting and service providers

STS therapists will conduct treatment groups in the schools. Supervision of therapists by agency supervisors will take place at the schools or agencies. Subsequent consultation for agency supervisors (on how to be effective supervisors) will be conducted remotely via Adobe Connect Pro Software. Adobe Connect Pro will be used for remote consultation purposes (i.e., video conferencing from CHOP to the agency location as needed for consultation of supervisor-to-therapist supervision). Research Electronic Data Capture (REDCap) is a web-based software supporting clinical and translational research databases. REDCap will enable the uploading and remote watching of treatment video sessions and therapist-to-supervisor supervision sessions, as well as serve as a digital space to collect fidelity rating forms. Both web-based platforms offer high levels of security. REDCap is HIPAA compliant, and Adobe Connect Pro uses the highest available encryption and security settings for online streaming services.

#### Training and consultation to agency clinical supervisors and therapists

**Fig. 1:**
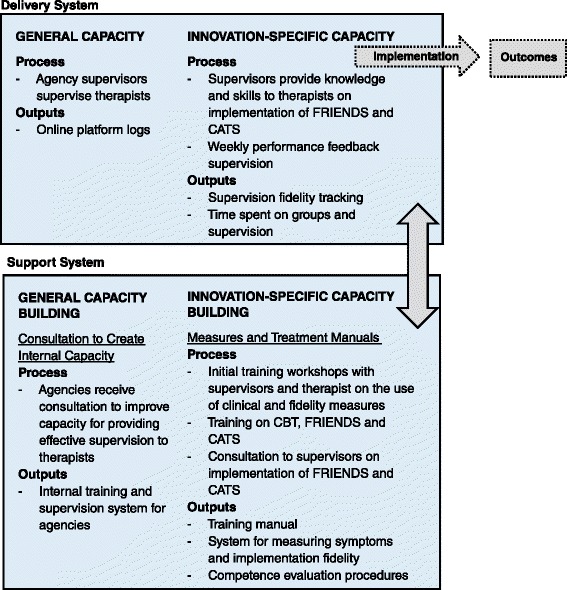
Interactive systems framework for dissemination and implementation

All activities related to the training and ongoing support for implementers are guided by the ISF [30] (see Fig. 1). The training and support procedures for therapists and supervisors in this proposal were informed by the ISF [30] (see Fig. 1) as well as the implementation studies in nontraditional settings [47–49], a review of training studies conducted by a member of our team [50], and a randomized clinical trial of different types of support for clinicians [26]. An initial workshop with therapists and supervisors will include a component describing the experimental design and trial procedures. Experts in the treatment of anxiety disorders will provide initial training to all therapists and supervisors. They will also provide remote consultation via Adobe Connect Pro to six supervisors in condition C. Agency supervisors will provide supervision to therapists in the implementation of FRIENDS (condition A) and CATS (conditions B and C).

##### Support system—general capacity building

The study will be conducted in the context of program general capacity building, specifically, training and consultation, to enable agency supervisors to become effective clinical supervisors [21]. Members of the research team will conduct 4 days of initial training in August of each year for all agency therapists (including alternates to protect against participant dropout) and supervisors. Supervisors will learn a competency framework for supervisors [51], strategies for identifying children who could benefit from the service (e.g., conducting in-service presentations to faculty on signs of anxiety in children) and how to access Adobe Connect Pro and REDCap while in the field (e.g., wireless access, using fidelity forms). They will also be trained on the use of fidelity monitoring and conducting performance feedback with therapists (see below). All supervisors will receive a supervision manual and a procedures manual.

##### Innovation-specific capacity building

Supervisors will learn how to prepare therapists for each treatment session and how to measure treatment fidelity and conduct performance feedback using fidelity forms. Therapists will be trained in using a screening instrument (SCARED) for the identification of potential participants. Therapists will be introduced to a competency model for CBT [52], which delineates generic therapeutic competencies (e.g., professional practice), CBT competencies (e.g., relevance of theory and research), and specific CBT techniques (e.g., managing negative thoughts). They will also learn about how to deal with implementation barriers (e.g., scheduling sessions, conducting exposure tasks) [53]. Therapists and supervisors from the six programs using FRIENDS and the 12 programs using CATS will be trained separately for activities related to each protocol. Trainers will follow training procedures used in other EBP dissemination studies in nontraditional settings [47, 54] and other training strategies found to be effective (i.e., active learning such as modeling and role playing) [50]. At the conclusion of the training, therapists and supervisors will be administered the Knowledge Test (KT) [55] to measure knowledge of concepts taught during the training. Participants who score below 80 % will be provided further training in the areas in which they scored low.

Two of the investigators who are licensed psychologists will provide ten consultation sessions in the first year via Adobe Connect Pro to six agency supervisors in the TT+ group (condition C; see Table 2). The consultation will be condensed into five sessions for returning supervisors in subsequent project years. The consultation sessions will center on helping the supervisor conduct supervision and will include how to perform performance feedback with therapists, implementation fidelity, and problem-solving implementation barriers and will answer supervisors’ questions about the supervision manual.

**Table 2:** Support provided to supervisors by condition

Train-the-trainer (groups A and B)	Train-the-trainer plus (group C)
• 4 days of initial training • Booster session 1 month prior to initial treatment session	• 4 days of initial training • 10 consultation sessions in the first year via online platforms

##### Delivery system—general capacity use

Supervisors in TT (conditions A–B) will receive a 2-h booster training session within a month prior to the first treatment session (see Table 2). The purpose of the booster session is to review key points from the training (e.g., conducting performance feedback) and to answer questions about any aspect of the supervision process. Through the initial training and subsequent booster session, STS programs will have the capacity to provide effective supervision to therapists, and therapists will have the capacity to use EBPs with fidelity. Supervisors will be expected to conduct supervision with therapists, and therapists will be expected to conduct FRIENDS or CATS groups with fidelity. However, we will not enforce these expectations so as to not introduce confounds to the data.

##### Innovation-specific capacity use

Supervisors will provide one 50-min supervision session for each treatment session the therapist conducts (12 for FRIENDS, 8 for CATS; see Table 3). The session is divided into two distinct categories: (a) group preparation (i.e., discussing referrals, preparing for upcoming session, engaging in problem-solving around implementation barriers) and (b) coaching (e.g., performance feedback).

**Table 3:** Support provided to clinicians (all conditions)

Provided by research team	Provided by agency supervisors
• 4 days of initial training • Video recordings of treatment main components	• 8 (group C) or 12 (groups A and B) coaching sessions: session preparation, self-reflection, goal setting, content, and process fidelity feedback

*Group preparation.* The main goal of this portion of supervision is to teach upcoming session content. Supervisors are to review content in the treatment manual (e.g., main session content; active learning activities), in order to prepare the therapist to conduct the upcoming session.

*Coaching.* The goal of this portion of supervision is to provide performance-based feedback on the previous treatment session. Prior to the session, the supervisor will log onto REDCap and watch a recording of the previous session, complete the fidelity data form, and identify clips for discussion using estimated timestamps. The supervisor will engage in a discussion of the therapist’s self-reflection. Then, the supervisor will provide the therapists with (a) fidelity data and (b) video clips from the previous session showing effective and ineffective implementation of FRIENDS/CATS. Fidelity data are provided to therapists with regard to *content* (i.e., the material they are supposed to cover in session) and *process* (i.e., how well they delivered the session). During these meetings, the therapists’ goal is to reach a high level of content and process fidelity (set at ≥80 % on the CFC and process fidelity set at an overall score of ≥3 on the PFC). All session video recordings and fidelity levels per session will be available to the therapists for on-demand examination for the duration of their participation in the study (i.e., 1 year).

Contamination between conditions (A, B, C) is expected to be minimal because there will be only one condition in each school. Experts will instruct supervisors to supervise therapists based only on what they have learned in the consultation sessions.

##### Compensation

Therapists will be provided a stipend for each supervision session attended with their supervisor. Supervisors will be provided a stipend at the conclusion of the treatment group. The stipends are based on projected time commitment to the project. Parents and teachers will receive a small stipend, and children will receive small gifts upon completing study measures.

#### School and child recruitment

At the beginning of each project year, we will conduct school-wide in-service training for teachers on recognizing warning signs of excessive anxiety in children. Research staff will do this in the first year, and STS staff will do this in subsequent years. Following the in-service, STS therapists will ask teachers to refer any child showing signs of anxiety to the STS program. The STS therapists will ask each parent and teacher of children referred to the STS program to complete a screening measure, SCARED. If a child meets the screening criteria on SCARED and becomes enrolled in the STS program at their school, research staff will ask the parents/caregivers to provide consent and the children to provide assent for a brief diagnostic evaluation to determine eligibility. The research aspect of the project will begin at this point. Children who meet criteria at the *positive* or *intermediate* levels will be invited to participate. No data will be collected on or about children whose parents do not consent to participate. However, these children will be allowed to take part in the groups if they choose to.

##### Attendance and missed sessions

Our procedures for ensuring child attendance to FRIENDS and CATS groups include strategic planning and attendance monitoring. For example, the therapist or agency staff will call the parent of each child in the group every other week to remind them of what the child is learning in the session and to review the child’s attendance thus far. Parents and teachers will be asked throughout the course of the treatment to encourage the child to attend the group. Supervisors will be instructed to discuss with therapists child attendance and problem-solve with them how to ensure continued participation for children who miss sessions. The components of the attendance strategy will be reviewed during the initial training of the therapists and supervisors. Continued implementation of the procedures will be reviewed in each supervision session between agency supervisors and therapists and between the expert consultants and supervisors. The strategy components will also be incorporated into the training and consultation manual. Therapists will work with individual children to cover missed material before the following session.

#### Treatment protocols

##### Friends for Life

FRIENDS [56] has been successfully implemented in several countries with children from diverse ethnic backgrounds [57, 58]. The original FRIENDS program consists of ten 70-min in-school sessions, two booster sessions, and four evening parent sessions. Due to the expected parent time and resource constraints, the four evening parent sessions are excluded. We reduced the session length to 40 min to be able to be conducted during one school period and included the two booster sessions in the protocol for a total of 12 40-min weekly sessions during the regular school day.

FRIENDS was developed based on the view that anxiety is a tripartite construct involving physiological, cognitive, and behavioral components [59]. Experts in childhood anxiety disorders have identified five essential components in CBT: psychoeducation, somatic management skills training, cognitive restructuring, exposure methods, and contingency management [12, 22].

##### CBT for Anxiety Treatment in Schools

The adapted protocol retains the core elements of evidence-based CBT for anxiety and the FRIENDS group format. We conducted planned adaptations to the protocol based on our collective experience with the protocol, two previous implementation studies [17, 60], and focus groups and qualitative interviews with stakeholders. We followed procedures developed by Lee and colleagues [21] and Bernal et al. [61], including surveying service providers and trainers regarding the appropriateness of FRIENDS for the target population. We conducted qualitative interviews with five consultants and six focus groups with school counselors who used FRIENDS. We made changes to the language (idioms, metaphors, words), cultural fit (cultural values), methods (session length (40 min), number of sessions (eight sessions)), and activities (in-session practices), which resulted in additions and substitutions in these areas while maintaining the five essential components of the treatment [12, 22]. This resulted in a briefer (eight sessions) and more feasible, engaging, and culturally appropriate protocol for urban under-resourced schools than the original FRIENDS.

#### Measures and assessment procedures

We are interested in measuring pre- to post-changes in effectiveness and implementation outcomes. The primary outcome measures organized by aim, construct, time point, informant, and method are presented on Table 4.

**Table 4:** Measurement instruments presented by aim, timepoint, method and informant

Aim	Construct	Instrument	Instrument characteristics	Timepoints	Method	Informant
Competence	Knowledge of CBT & treatment of anxiety	Knowledge Test (KT) [55]	20-item questionnaire rated on a true/false or multiple choice format	Initial training	Self-report	Therapist
Sample description	Family characteristics	Demographic information	Age, grade, gender, race/ethnicity, and socioeconomic status	Pre- diagnostic evaluation	Self-report	Parents
Screening	Anxiety disorders	Screen for Child Anxiety Related Disorders (SCARED) [43]	41-item, 3-point scale (0 = not true or hardly ever true to 2 = very true or often true) organized around five scales and a Total Score	Pre- diagnostic evaluation	Child behavior rating	Parents & teachers
Aim 1-2	Effectiveness Child psychopathology	Anxiety Disorders Interview Schedule for Children – DSM-5, Parent Version (ADIS-5-P) [44, 45]	Semi-structured psychiatric interview. English & Spanish versions. DSM-V diagnoses, severity, and comorbidity. Clinical judgment is required to determine clinical diagnosis (Clinician Severity Rating; CSR ≥4) and identify those at risk (CSR=3)	Pre- & post- treatment	Child behavior report	Parents
Aim 1	Clinical Global Impression Severity	Clinical Global Impression - Severity CGI-S [71]	Global score based on a 7-point scale (1 = normal, not at all ill to 7 = among the most extremely ill), with lower scores indicating less severity	Pre- & post- treatment	Functional impairment rating	Diagnostician
Aim 1	Clinical Global Impression Improvement	Clinical Global Impression - Improvement CGI-I [71]	Global improvement score based on a 7-point scale (1 = very much improved to very much worse); assigned at post-treatment for the primary diagnosis	Post- treatment	Functional impairment rating	Diagnostician
Aim 1	Global impairment	Children’s Global Assessment Scale (CGAS) [72]	Children ages 4–16 years. 1–100 scale reflecting level of child’s functioning during a specified time period	Pre- & post- treatment	Functional impairment rating	Diagnostician
Aims 1-2	Anxiety symptoms	Multidimensional Anxiety Scale for Children - 2^nd^ Edition (MASC 2) [73]	50-item, 4-point rating scale (0 = never to 3 = often). Sensitive for measuring treatment effects [73]	Pre- & post- treatment	Child behavior rating & Self-report	Parents & children
Aims 1-2	Academic competence	Academic Competence Evaluation Scales (ACES) [74]	Ratings for Reading/Language Arts and Math and academic enablers (i.e., engagement in academic activities and motivation to achieve). Sensitive for measuring intervention effects [74–77]	Pre- & post- treatment	Child academic performance rating	Teachers
Aim 2	Implementation Content fidelity	Content Fidelity Checklist (CFC)	Yes/no rating scale to indicate whether or not a therapist covered a particular component of the treatment	Ongoing	Video coding	Independent coding of therapist behavior
Aim 2	Process fidelity	Process Fidelity Checklist (PFC) [47]	12-item, 5-point scale (0 = not at all, to 4 = very often)	Ongoing	Video coding	Independent coding of therapist behavior
Aim 2	Content & process fidelity	Supervision Content & Fidelity Measure (SCFM)	Supervision content and process as detailed in the training manual, e.g., prepared for next session; Demonstrated empathy and provided positive reinforcement	Ongoing	Self-report	Supervisor
Aim 2	Content & process fidelity	Consultation Content & Process Measure (SCPM)	Consultation content and process as detailed in the training manual, e.g., prepared for next session; Demonstrated empathy and provided positive reinforcement	Ongoing	Self-report	Consultant
Aim 2	Appropriateness & acceptability	Qualitative interviews	Interviewers will follow a script to ask questions of supervisors and therapists, e.g., *“How acceptable is the level of support you received for the implementation of the FRIENDS protocol?”; “Was the supervision you received from your agency’s supervisor appropriate for running groups with children?”; “Was the consultation you received from the research team appropriate for preparing you to conduct supervision with STS therapists?”*	Post-treatment	NVivo coding [78] of interview transcriptions	Therapists & supervisors
Aim 1-2	Cost	Modified DATCAP interview [79, 80]	Therapist time, trainer time, expert supervision, time implementing the program, and cost of materials used in the implementation of the program	Ongoing	Multi-method	Administrators
Aim 3	Moderators Behavioral intentions	Behavioral Intentions (BI) [36, 35]	Two items, 7-point scale (1 = very unlikely, to 7 = very likely), e.g., “*How likely is that you will participate in supervision for the implementation of Friends for Life in the school setting?*”	Pre- implementation	Self-report	Therapists
Aim 3	Mediators Service interruptions	Service Interruption Coding (SIC)	Therapist availability for treatment session and supervision: 1) Projected length of the session in minutes minus time spent on interruptions (e.g., answering a phone call, talking about unrelated topics); 2) Number of interruptions; 3) Total number of supervision / consultation and treatment sessions per group	Treatment sessions & supervision	Video coding	Independent coders
Aim 3	Reflection on Process; Professional Flexibility; Newly Created Professional Activities; Role Interdependence	Index of Inter-professional Team Collaboration for Expanded School Mental Health (IITC-ESMH) [81]	26-item, 5-point scale (1 = never, to 5 = always)		Self-report	School team - School & agency employees

### Study aims

*Aim 1*. To compare pre- to post-treatment effectiveness of CATS to FRIENDS and to compare the total cost between CATS and FRIENDS.

We hypothesize that (1a) children in CATS will show equivalent-to-better clinician severity rating (diagnostic status), global functioning, symptom severity, impairment, and academic competence compared to children in FRIENDS; (1b) the total cost of CATS will be less than the cost of FRIENDS; and (1c) CATS will be superior to FRIENDS by incurring less cost and showing either equivalent or better clinician severity rating than FRIENDS.

Hypothesis (1a) will be tested by calculating the 95 % confidence limits (the width of the limit = |4.20|) for the differences in diagnostic status, global functioning, symptom severity, impairment, and academic competence (child outcomes) from pre to post between children receiving FRIENDS and children receiving CATS. Differences will be calculated as CATS changes − FRIENDS changes. If the calculated differences are within the 95 % limit, then we will conclude that the two interventions have an equal impact or effect on child outcomes. If any of the differences in child outcomes are larger than the upper limit, then we will conclude that CATS is better than FRIENDS. The limit of |4.20| was chosen based on the distribution of the reported mean effect sizes associated with FRIENDS [13].

Hypothesis (1b) will be tested by calculating the 95 % confidence interval (CI) for the difference in total cost. Differences in total cost will be considered significant when the 95 % CI exclude zero. Costs will be assessed by use of generalized linear models (GLM). Links and families for the GLM will be empirically fit to the data using diagnostic tests including the modified Parks test, Pregibon link test, Hosmer-Lemeshow test, and Pearson’s correlation test [62].

Hypothesis (1c) will be tested by using information about the difference in effect and the difference in cost to calculate the cost-effectiveness ratio (difference in cost/difference in effect) and the 95 % CI for this ratio. A confidence interval that excludes zero indicates that the intervention reduces cost and improves the CGI-S rating that is completed by a clinician as part of the ADIS-P semi-structured interview with the parent (i.e., the percentage of children experiencing an improvement). If the upper 95 % confidence limit includes zero, the result would indicate that we cannot rule out with 95 % confidence that the therapy is improving health at an increased cost.

*Aim 2*. To compare two implementation strategies, TT and TT+, for the implementation of CATS.

(2a) Our primary hypothesis is that TT+ will yield higher therapist content fidelity scores compared to TT. (2b) TT+ will also show better process fidelity and higher perceived acceptability compared to TT. We also expect that (2c) the TT strategy will lead to acceptable levels of therapist fidelity (content fidelity ≥80 %; process fidelity ≥4 on a 1–5 scale, 1 = not at all, 5 = very often) and (2d) TT+ will be more cost effective than TT because TT+ will yield better child outcomes (i.e., lower CGI-S scores) with the same number of treatment weeks (i.e., 8 weeks). In addressing this aim, comparison between the TT and TT+ will be done using a two-sided two-sample *t* test. Therapist content fidelity means will be calculated with its 95 % confidence interval. Similar analysis will be done regarding process fidelity. For hypothesis (2d), we will compare the difference in the average cost per therapist and the difference in average content fidelity (i.e., difference in the percentage of checklist components covered by the therapist). Measures of effectiveness will be derived from the *clinical* analysis of the trial data. Standard errors will be derived from a nonparametric bootstrap. Point estimates for the cost-effectiveness ratios will be based on the point estimates for the difference in costs and outcomes. Standard errors and the correlation of the difference in cost and QALYs will be derived from a nonparametric bootstrap. Confidence intervals for the cost-effectiveness ratio as well as an acceptability curve will be derived using the point estimates of the differences, their standard errors, and the correlation between the differences [62]. Acceptability curves will allow agency administrators to judge whether the gains in children’s outcomes/fidelity are sufficient to justify any increases in cost.

Secondary outcomes for therapists will include process fidelity (i.e., difference in the five-point rating of therapist competence). One set of these comparisons will be made for FRIENDS with TT vs. CATS with TT arms (i.e., is CATS cost-effective compared to FRIENDS); a second set will be made for CATS with TT+ vs. CATS with TT (i.e., is CATS with TT+ cost-effective compared to CATS with TT). The numerator of the cost-effectiveness ratio will be the difference in costs between the groups (e.g., the costs of CATS with TT+ minus the costs of CATS with TT); the denominator will be the difference in outcome (e.g., the change in CSR for CATS with TT+ minus the change for CATS with TT).

To test the second part of hypothesis (2b), we will conduct a structured content analysis [63] for perceived acceptability and appropriateness. The unit of analysis is each interview. We will create a case-by-variable matrix from the codes. The matrix will have an interviewee identifier number, type of support (i.e., TT, TT+), school, agency, subject demographic information, and codes. The code cells will contain the number of times the participant emitted words or phrases pertaining to perceived acceptability and appropriateness of the support they received. Once acceptability and appropriateness are defined for each interview, percentages for acceptability (percentage acceptance) and appropriateness (percentage appropriate) out of the total number of words will be calculated. Comparison of percentage acceptance and percentage appropriate between TT and TT+ will be conducted using the Wilcoxon sum-rank test.

*Aim 3*. To test mediators and moderators of the type of support on therapist fidelity.

**Fig. 2:**
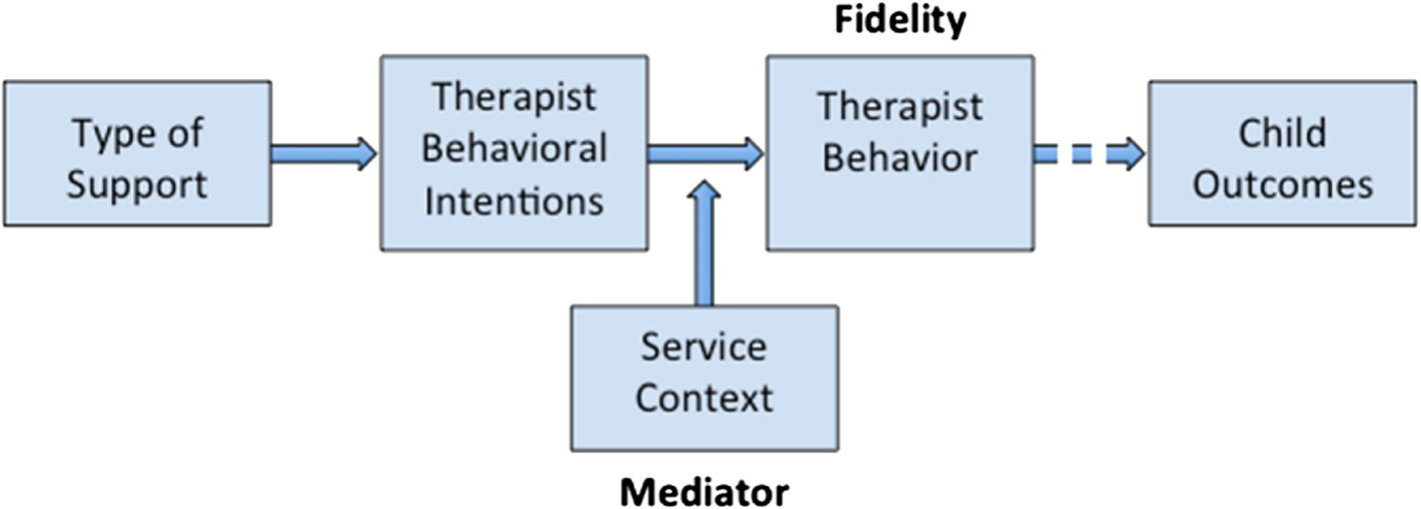
Moderators and mediators of intervention effects on implementation fidelity

We hypothesize that therapists’ strength of intentions to conduct groups with children and take part in supervision will moderate the relationship between actual level of treatment fidelity and the type of implementation support they receive (see Fig. 2). We are studying the role of intentions because this construct has been shown to be a proximal determinant of treatment fidelity when practitioners have the ability to act on their intentions [34, 35].

In the current study, moderation occurs when the strength of the association between a predictor, type of support (TT or TT+), and fidelity (dependent variable) is influenced by a therapist intention (moderator). Moderation will be assessed using multiple regression analysis as described by Preacher et al. [64], for analyzing moderation effects. In a regression model setting, in step 1, type of support and therapist will be entered first to predict fidelity. In step 2, the interaction term of therapist intention with type of support will be entered into the regression model. If the slope of the interaction terms is found to be significantly predicting fidelity, then we will conclude that the therapist intention moderates the effect of type of support on fidelity.

We also expect that the service context (including interdisciplinary collaboration and service disruptions) will mediate the relationship between the therapists’ fidelity and their intentions to perform the EBPs. We are studying the role of service context (i.e., frequency of interdisciplinary collaboration, environmental intrusions affecting the ability of therapists to fully participate in supervision and conduct treatment groups) because it may facilitate or impede the therapists’ ability to act on their intentions [36, 65]. Therefore, we will also test whether service context mediates the relationship between therapists’ intentions and actual treatment fidelity.

The mediation analyses will examine the direct effect of therapist intention (*I*) on fidelity (*F*) and the indirect effect of *I* on *F* through service context (*S*). Four conditions must be satisfied for service context to be a mediator: (1) therapist intention is significantly associated with fidelity. Using a simple regression model with *F* being the dependent variable and *I* as the independent variable. (2) *I* is significantly associated with *S*. Using simple regression model with *S* being the dependent variable and *I* as the independent variable. (3) *S* is significantly associated with *F* (after controlling for *I*). Using a multiple regression model with *I* and *S* being a predictor and *F* as the dependent variables. (4) The impact of *I* on *F* is significantly reduced after controlling for *S*. We will test the four conditions above using three regression models. In planning and performing the analyses related to moderation and mediation, we will utilize PROCESS, a modeling tool freely available for SPSS and SAS. The many features of PROCESS integrate many existing functions and published statistical tools for analyzing mediation and moderation [66]. The SAS [67] and SPSS [68] software will be used for data analysis.

### Statistical considerations

The unit of randomization at the school level may potentially produce differences among students’ baseline measurements such as demographic information, diagnostic status, academic functioning, and therapists’ level of experience among groups. Assessment of such differences will be examined using *t* tests or the nonparametric Mann-Whitney test for two independent groups. Confirmed baseline differences between study arms regarding baseline student’s measurements will be included as covariates in the proposed pertinent statistical analysis. It is understood that outcomes related to a student in a school/class may be correlated with outcomes for other student within the same school/class (nested effects). As such, the correlation (intraclass correlation) among students in a school/class will be calculated and the expected nested effects will be considered within the scope of addressing the proposed aims. We will conduct analyses using SAS Proc mixed (the mixed effect approach) (or the Proc GENMOD for the GEE approach), in which students are considered to be nested within schools/classes. The mixed effects model or the GEE approach will be used in the analysis of pre-/post-changes between the groups. Time (pre/post), interventions (or implementation), and the interaction term of time*intervention (or implementation) are the effects of interest. We may consider simplifying the analysis when it is appropriate by calculating the change scores between pre and post and testing for means differences using *t* tests for two independent groups (or the Mann-Whitney test). Also, if needed, the analysis of variance and covariance might be utilized to compare pre-/post-measurements between groups adjusting for pre-measures and any other potential covariates.

### Power analyses

#### Children

It is anticipated that a sample size of 240 evaluable children are available to address aim 1 (120 per treatment protocol). The published [13] reported effect size of FRIENDS has been estimated to equal to 0.56 (Cohen’s *d*). In a pilot study, we found that the change in symptom level had an effect size of 0.63. Therefore, in testing for equivalence of FRIENDS and CATS, using two one-sided tests with sample sizes of 120 in FRIENDS and 120 in CATS achieves 81 % power at a 5 % significance level when the true difference between the means is 0 (null hypothesis), the standard deviation of the differences in means is assumed to be 11, and the equivalence limits are −4.20 and 4.20.

#### Therapists

To compare TT to TT+ for the implementation of CATS (aim 2), 30 therapists in TT and 30 in TT+ achieve 86 % power to detect a difference of 8 % between the null hypothesis that both group means are 80 % (content fidelity) and the alternative hypothesis that the mean of group TT is 80 % with the estimated group standard deviations of 10 and 10 (an estimated effect size = 0.8) and with a significance level (alpha) of 0.05 using a two-sided two-sample *t* test. Similarly, group sample sizes of 30 and 30 achieve 84 % power to detect a difference of 0.7 in process fidelity between the null hypothesis that both group means are 4 and the alternative hypothesis that the mean of group TT+ is 4.7 with the estimated group standard deviations of 0.9 and 0.9 (an estimated ES = 0.78) and with a significance level (alpha) of 0.05 using a two-sided two-sample *t* test.

## Discussion

The justification for the effectiveness portion of the trial (group A vs. B) is based on the fact that most EBPs need to be adapted to improve fit to context but that the new version of the treatment must be compared to the original version to determine effectiveness. The justification for the concurrent implementation portion of the trial (group B vs. C) rests on the fact that publicly funded agencies need cost-effective, research-based implementation strategies to justify widespread adoption of EBPs. The use of the hybrid trial approach is justified by the fact that the hybrid approach shortens the translation process [41].

### Public health impact

This study has the potential to demonstrate that agency therapists and supervisors who have had little to no prior exposure to EBPs can implement an anxiety disorder EBP with fidelity. The use of group therapy can contribute to lowering service disparities in urban schools. Comparisons of the two implementation strategies (internal supervision with or without consultation for supervisors) would provide large urban mental health systems with data to make decisions about adoption of EBPs [40]. The study is consistent with the goals of the Affordable Care Act in that it would generate information on the implementation of effective practices that optimize delivery of mental health care to traditionally underserved communities [69].

### Innovation

This study offers a number of bold innovations. This is the first study, that we are aware of, that conducts a hybrid effectiveness-implementation trial of GCBT in nontraditional settings. Comparing both the effectiveness of an adapted treatment to its original and the implementation outcomes of train-the-trainer from those of an adapted version in the same trial is innovative. The assessment of mediators and moderators of the two implementation strategies on therapist fidelity as well as the assessment of comparative cost and cost-effectiveness of implementation strategies in the co-location service context is highly innovative [40]. The measurement of clinical *and* academic outcomes in the context of an effectiveness-implementation trial is also innovative [32].

### Limitations

Although there are a number of strengths to the proposed work including the hybrid effectiveness-implementation trial, use of frameworks to guide the proposed work, and focus on an underserved population, there are also limitations. First, some of the measures in the proposed work are investigator created, which is often the case in implementation science trials given the nascence of the field [70]. Second, because of the effectiveness nature of the trial, there were some decisions that had to be made to ensure that youth participants met criteria for an anxiety disorder. Specifically, we are using the ADIS-5-P for diagnostic purposes which is likely not feasible in community settings outside of the context of a grant. Additionally, the therapists will likely engage in more intensive attendance monitoring to ensure youth attendance than would occur outside the context of a research study. Finally, although creating internal capacity is a worthwhile avenue for research, there are still indicators that supervisor turnover is an issue to be grappled with (see Beidas et al., in press).

### Conclusions

This study has the potential to show that masters-level therapists from public mental health agencies providing services in schools can implement EBPs with high levels of fidelity, provided that they receive ongoing evidence-based supervision from their own clinical supervisors. The implications of this project are generalizable to other evidence-based practices and have the potential to significantly move the field forward. Gathering information about the cost-effectiveness of the two implementation strategies (TT vs. TT+) will provide important data for future implementation efforts when policymakers and administrators are deciding between paying for external consultation and creating internal capacity from within to support implementation of EBPs.

### Trial status

ClinicalTrials.gov identifier: NCT02651402. The study was approved by the Committee for the Protection of Human Subjects of the Children’s Hospital of Philadelphia (IRB 15-012311), the Office of Research and Evaluation (ORE) Research Review Committee of the School District of Philadelphia (IRB 2015-09-381), and by the City of Philadelphia (IRB 2015-46).

## Abbreviations

CATS, CBT for Anxiety Treatment in Schools; CBT, cognitive behavioral therapy; FRIENDS, Friends for Life; GAD, generalized anxiety disorder; GCBT, group cognitive behavioral therapy; SAD, separation anxiety disorder; SoAD, social anxiety disorder; STS, school therapeutic services; TT, train-the-trainer; TT+, train-the-trainer plus
